# Graphene/PtSe_2_/Ultra-Thin SiO_2_/Si Broadband Photodetector with Large Responsivity and Fast Response Time

**DOI:** 10.3390/nano15070519

**Published:** 2025-03-29

**Authors:** Qing-Hai Zhu, Jian Chai, Shi-Yu Wei, Jia-Bao Sun, Yi-Jun Sun, Daisuke Kiriya, Ming-Sheng Xu

**Affiliations:** 1 College of Integrated Circuits, State Key Laboratory of Silicon and Advanced Semiconductor Materials, and Zhejiang Key Laboratory of Advanced Micro-Nano Transducers Technology, Zhejiang University, Hangzhou 310027, China; 2 College of Information Science & Electronic Engineering, Zhejiang University, 38 Zheda Road, Hangzhou 310027, China; 3 Department of Basic Science, and Department of Integrated Sciences, The University of Tokyo, Tokyo 113-8654, Japan

**Keywords:** two-dimensional PtSe_2_, graphene contact, passivation layer, carrier collection, broadband photodetector

## Abstract

Burgeoning two-dimensional (2D) materials provide more opportunities to overcome the shortcomings of silicon-based photodetectors. However, the inevitable carrier loss in the 2D material/Si heterojunction has seriously hindered further improvement in responsivity and detection speed. Here, we propose a graphene/PtSe_2_/ultra-thin SiO_2_/Si photodetector (PD) with multiple optimization mechanisms. Due to the fact that photo-generated carriers can travel in the graphene plane toward the Au electrode, the introduction of a top graphene contact with low sheet resistance provides a carrier collection path in the vertical direction and further restricts the carrier recombination behavior at the lateral grain boundary of PtSe_2_ film. The ultra-thin SiO_2_ passivation layer reduces the defects at the PtSe_2_/Si heterojunction interface. As compared to the counterpart device without the graphene top contact, the responsivity, specific detectivity, and response speed of graphene/PtSe_2_/ultra-thin SiO_2_/Si PD under 808 nm illumination are improved to 0.572 A/W, 1.50 × 10^11^ Jones, and 17.3/38.8 µs, respectively. The device can detect broad-spectrum optical signals as measured from 375 nm to 1550 nm under zero bias. The PD line array with 16-pixel units shows good near-infrared imaging ability at room temperature. Our study will provide guiding significance for how to improve the comprehensive properties of PDs based on 2D/Si heterostructure for practical applications.

## 1. Introduction

As the core component of photoelectric information conversion, the performance improvement of photodetectors (PDs) is directly related to the progress in many fields, such as optical communication, medical imaging, sensing detection, and even smart cars [1,2,3]. Bulk silicon plays an indispensable role in PDs due to its abundant reserves, cheap cost, and advanced manufacturing process [4]. Combining silicon with other materials possessing different energy band structures (such as Ge, MgSi_2_, GaAs, and perovskite) can tailor the barrier at heterojunction interfaces, thus effectively regulating the carrier transport and enhancing the photoelectric conversion efficiency [5,6,7]. However, these heterojunction devices usually suffer from the shortcomings of lattice mismatch, large energy consumption, expensive prices, and poor stability [8,9]. With the rapid upgrading of photoelectric technology, it is urgent to design feasible strategies to enhance the responsivity of photodetectors and optimize the collection efficiency of carriers.

Recently, 2D materials with fascinating characteristics such as easy integration, high mobility, and strong light–matter interaction, have been widely used in the manufacturing of silicon-based heterojunction photodetectors [10,11]. Unfortunately, most 2D materials have some shortcomings that cannot be ignored. For example, due to the poor optical absorption and zero band gap of graphene, the graphene/Si Schottky junction usually suffers from low responsivity and a large dark-state current [12]. Black phosphorus (BP) with a narrow band gap can extend the effective detection range of Si-based PD to mid-infrared light [13]. However, the poor stability of BP in the atmosphere makes the preparation and storage of devices difficult [14]. The large band gap of many 2D transition-metal dichalcogenides (TMDs) also limits their application prospects in the field of broadband photodetection [15,16,17]. Two-dimensional platinum selenide (PtSe_2_), as a typical noble metal dichalcogenide material, has the advantages of an adjustable band gap based on its thickness (from 1.2 eV to 0 eV), high carrier mobility, good stability, and easy synthesis [18,19,20]. The unique electronic structure and topological surface state of PtSe_2_ enable carriers to be transported quickly, effectively reducing the recombination of electron–hole pairs [21,22]. By combining 2D PtSe_2_ material with Si substrate, a strong built-in electric field can be formed at the interface of the heterojunction, thus realizing the self-powered detection of optical signals in a wide wavelength range. For example, Xie et al. successfully fabricated a self-powered PtSe_2_/Si vertical heterojunction photodetector by directly growing PtSe_2_ on silicon material [23]. The introduction of PtSe_2_ film extended the effective detection range of Si-based PD to 1550 nm; the PtSe_2_/Si device obtained a maximum responsivity of 0.52 A/W and rise/fall times of 55.3/171 μs for 808 nm illumination at zero bias. Ma et al. heterogeneously integrated PtSe_2_ material with a pyramid-shaped microstructure Si substrate by a traditional thermal-assisted selenization method; the responsivity of this self-powered device was improved to 0.567 A/W under 810 nm illumination [24]. The 8 × 8 PtSe_2_/pyramid-Si PD array exhibited good uniformity and imaging application ability. Ye et al. demonstrated that the dark current of self-driven PtSe_2_/Si PD can be decreased without suppressing the photocurrent by using a SiO_2_ insulating layer with appropriate thickness at the PtSe_2_/Si interface [25]. Despite the progress, the transport pathway and capture efficiency of carriers in heterojunction systems need optimization to improve the device’s comprehensive performance. In most cases, the currently prepared 2D materials such as PtSe_2_ are polycrystalline and there are lots of grain boundaries and other types of defects, which influence carrier transport and slow down the response time of the devices. We believe that the photodetection performance of PtSe_2_/Si vertical PDs would be further enhanced by optimizing the collection pathway of photogenerated carriers and improving the quality of heterojunction interfaces.

Herein, we design an architecture of graphene/PtSe_2_/ultra-thin SiO_2_/Si for two-terminal PD, where the graphene (Gr) is used as the top contact with PtSe_2_ to collect photogenerated carriers of the device and the ultra-thin SiO_2_ plays the role of the interface passivation layer. The graphene layer with low sheet resistance can facilitate the transport of photogenerated carriers in a vertical direction, thus suppressing the recombination behavior at the lateral PtSe_2_ grain boundary and enhancing the collection efficiency of carriers. The exploitation of these strategies enables our Gr/PtSe_2_/ultra-thin SiO_2_/Si self-powered device to exhibit satisfying photodetection characteristics in the wavelength range of 375 nm to 1550 nm. Under the near-infrared illumination of 808 nm, the device obtains a high responsivity of 0.572 A/W, a large specific detectivity of 1.50 × 10^11^ Jones, and a fast response time of 17.3/38.8 μs. Furthermore, the PD line array with excellent homogeneity and repeatability shows good imaging application potential for near-infrared light at room temperature. Our study adopting the synergistic effect of various strategies promotes the development of high-performance, broadband, and self-powered photodetection technology.

## 2. Experimental

### 2.1. Material Synthesis and Device Fabrication

The n-type silicon substrates (resistivity: 1–10 Ω cm^−1^) with a 300 nm SiO_2_ insulating layer were used in the manufacture of heterojunction PDs. Firstly, the Si window with a size of 200 × 200 μm^2^ was defined by utilizing photolithography technology, and the sample was soaked in a buffered oxide etchant (HF:NH_4_F = 1:6) for about 3 min to remove the redundant SiO_2_ insulating layer. Then, the substrate was placed on a heating plate at 105 °C for 90 s to form a dense SiO_2_ passivation layer with a thickness of about 3.5 nm on the surface of the silicon window. Similarly, the growth region of PtSe_2_ material on a pre-prepared Si substrate was defined by ultraviolet photolithography technology. A Pt layer with a thickness of about 7 nm was deposited by a magnetron sputtering method (sputtering at a power of 20 W for about 60 s). Next, 900 mg selenium powder (99.99%) and the prepared Pt/ultra-thin SiO_2_/Si sample were placed in the upstream and downstream regions of the tube furnace, respectively. After 10 min, the temperature of the two heating regions reached 230 °C and 400 °C, respectively. During the process of thermal-assisted selenization, a vacuum pump was used to maintain the low-pressure environment in the quartz tube, and the Ar/H_2_ mixed gas (90/10 sccm) was continuously introduced as the carrier gas. After reacting for 90 min, the deposited Pt layer was completely selenized into the PtSe_2_ material. The PtSe_2_/ultra-thin SiO_2_/Si substrate was not taken out until the temperature was cooled to room temperature. Next, a Cr/Au electrode (5/50 nm) was deposited on the outer edge of the PtSe_2_ film by magnetron sputtering and lift-off methods. By using standard wet transfer technology (i.e., using PMMA as the supporting layer of graphene and etching the copper foil substrate with ammonium persulfate aqueous solution), graphene synthesized via the CVD method was transferred to the surface of PtSe_2_/ultra-thin SiO_2_/Si structure. Next, the redundant graphene material was removed by photolithography and an oxygen plasma etching process, thus realizing the delicate contact between the graphene layer and the device window. Finally, In-Ga alloy was coated on the back of the Si wafer to act as the bottom electrode. 

### 2.2. Characterization

The lattice structure of the PtSe_2_ film was analyzed by X-ray diffraction (XRD, Bruker, Berlin, Germany) and selected area electron diffraction (SAED, JEM 2100F, Amagasaki, Japan). Atomic force microscopy (AFM, Bruker Dimension ICON, Berlin, Germany) was performed to measure the thickness of the PtSe_2_ film. The thickness of the SiO_2_ passivation layer was evaluated by an ellipsometer (Uvisel, Horiba, Japan). The microstructure and morphology of the as-grown PtSe_2_ film were observed by scanning electron microscopy (SEM, Hitachi S4800, Tokyo, Japan) and transmission electron microscopy (TEM, JEM 2100F, Kitakyushu, Japan). Raman spectra of PtSe_2_ and graphene materials were obtained by using a 532 nm laser as excitation light on a Raman spectrometer (Horiba HR EVO, Kyoto, Japan). Ultraviolet photoelectron spectroscopy (UPS, Shimadzu Axis Supra, Manchester, UK) was employed to measure the secondary-electron cut-off region of PtSe_2_ film. The absorption spectra of PtSe_2_, Si, and PtSe_2_/ultra-thin SiO_2_/Si heterojunction were characterized by a UV–Vis–NIR spectrometer (Hitachi U-4100, Tokyo, Japan). The surface structure and morphology of the devices were observed using an optical microscope (Nikon Optiphot 200, Tokyo, Japan). The metal layers were deposited via a magnetron sputtering system (Discovery-635, New York, NY, USA). The photoelectric test platform for our heterojunction devices was composed of 375 nm, 532 nm, 808 nm, 940 nm, and 1550 nm lasers and a semiconductor parameter analyzer (FS480, Suzhou, China). In order to record the time-dependent photoresponse, the laser source was modulated by a signal generator (RIGOL DG5100, Suzhou, China) to output periodic optical signals.

## 3. Results and Discussion

**Figure 1:**
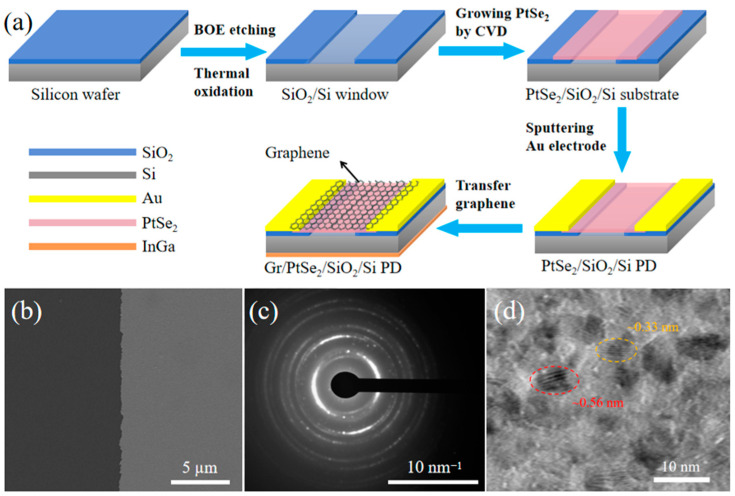
(**a**) Schematic diagram of the preparation of PtSe_2_/ultra-thin SiO_2_/Si PDs with and without graphene top electrode. (**b**) SEM image of PtSe_2_ film (right half area) grown on the silicon substrate. (**c**) SAED pattern of PtSe_2_ film. (**d**) TEM image of PtSe_2_ material with high-resolution magnification.

The schematic diagram of the preparation of PtSe_2_/ultra-thin SiO_2_/Si PDs with and without a graphene top electrode is exhibited in Figure 1a. Briefly, the Si substrate with about 300 nm of an insulating layer was etched by buffer oxide etchant and then thermally oxidized, thus exposing the n-type Si windows with an ultra-thin SiO_2_ passivation layer. In Figure S1, the refractive index (*n*) and extinction coefficient (*k*) values of the SiO_2_ film measured by ellipsometer demonstrate that a high-quality SiO_2_ passivation layer was formed on the bare Si windows [26], and its fitting thickness is about 3.5 nm. A dense passivation layer is usually beneficial to reduce defects at the heterojunction interface [27]. Then, the PtSe_2_ material was synthesized on the Si windows with the SiO_2_ passivation layer by a thermal-assisted selenization method. The Cr/Au electrode was deposited on the outer edge of the PtSe_2_ film by magnetron sputtering, obtaining the PtSe_2_/ultra-thin SiO_2_/Si heterojunction PD. Finally, graphene material was transferred to the top of the heterojunction to promote carrier collection. As seen from the SEM image in Figure 1b, the PtSe_2_ film synthesized on the Si substrate is continuous and quite uniform. Moreover, a number of diffraction rings with different spacing are observed by selected area electron diffraction (SAED) technology, which suggests that the PtSe_2_ film is a polycrystalline structure (Figure 1c). As shown in Figure 1d, the high-resolution TEM image of PtSe_2_ material shows lattice stripes with different orientations and spacing (such as ~0.56 nm and ~0.33 nm, corresponding to the (001) and (100) crystal planes of PtSe_2_, respectively), which further reveals that the polycrystalline film is composed of different PtSe_2_ domains. The grain boundaries between adjacent PtSe_2_ domains have a noticeable influence on carrier transport [28]. Therefore, it is of great significance to exploit the graphene top contact to collect photo-generated carriers in a vertical direction and make the carrier travel in the graphene plane toward the Au electrode.

**Figure 2:**
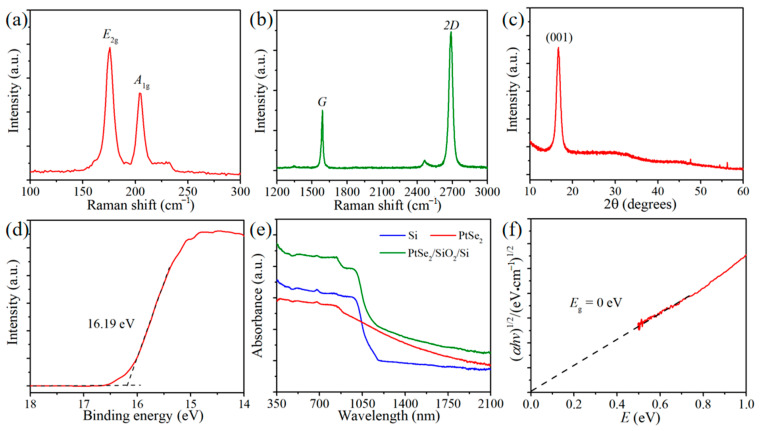
Raman spectra of (**a**) PtSe_2_ film and (**b**) graphene. (**c**) XRD pattern and (**d**) secondary-electron cut-off region of the PtSe_2_ thin film on the silicon substrate. (**e**) UV–Vis–NIR absorption spectra of Si, PtSe_2_, and PtSe_2_/ultra-thin SiO_2_/Si heterojunction. (**f**) Tauc plot of the 2D PtSe_2_ film.

Similar to other 2D TMDs, PtSe_2_ has a typical sandwich-like molecular structure [29]. The strong Raman peaks of our PtSe_2_ film at about 176 cm^−1^ and 207 cm^−1^ correspond to its *E*_g_ in-plane and *A*_1g_ out-of-plane vibration modes, respectively (Figure 2a) [30]. The intensity ratio of the 2*D* peak at ~2691 cm^−1^ to that of the *G* peak at ~1590 cm^−1^ of the Raman spectrum of the graphene layer is about 2.34 (Figure 2b), suggesting its monolayer nature [31]. The negligible *D* peak at about 1349 cm^−1^ further indicates that the graphene is of high quality with low defect density [32]. In Figure S2, the Raman spectrum of the graphene/PtSe_2_ heterostructure contains the characteristic peaks of PtSe_2_ and graphene materials, which indicates that the graphene/PtSe_2_ interface prepared by transferring graphene to the surface of the PtSe_2_ film is of good quality. Furthermore, the sheet resistance of graphene film is measured to be about 450 Ω sq^−1^, which is smaller than that of PtSe_2_ film (~1030 Ω sq^−1^), implying that the use of graphene as the top contact is beneficial to the carrier transport. As shown in Figure 2c, XRD was utilized to further analyze the crystal structure of the material. The obvious peak at 2*θ* = 16.8° is assigned to the dominant (001) crystal plane of PtSe_2_ [33]. Based on the EDS mapping in Figure S3, the uniform and consistent distribution of Pt and Se elements implies that the metal precursor had been completely transformed into PtSe_2_ compound by the CVD process. As shown in Figure S4, the thickness of the PtSe_2_ film analyzed by atomic force microscopy (AFM) is about 28 nm. According to the secondary-electron cut-off region in Figure 2d, the Fermi energy level (*E*_F_) of the PtSe_2_ film can be calculated by the following formula [34]: *E*_F_ = *hν* − *E*_cutoff_ = 21.22 eV − 16.19 eV = 5.03 eV. The UV–Vis–NIR absorption spectra of Si, PtSe_2_, and the PtSe_2_/ultra-thin SiO_2_/Si heterojunction are displayed in Figure 2e. Due to the inherent band gap of about 1.12 eV, the effective absorption spectrum range of Si is limited to 1100 nm [35]. It can be found that the introduction of the PtSe_2_ film enhances the light absorption ability of heterojunction while expanding the detection range of Si beyond 1100 nm. As shown in Figure 2f, the fitting Tauc plot reveals that the band gap of our PtSe_2_ film is about 0 eV, which is consistent with related reports [36].

**Figure 3:**
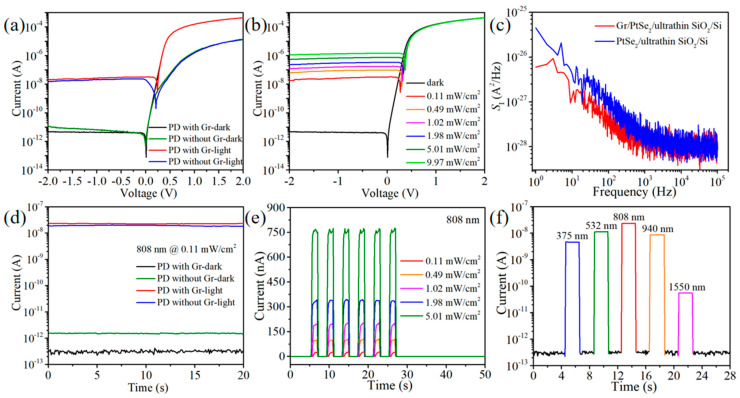
(**a**) *I*-*V* curves of the PtSe_2_/ultra-thin SiO_2_/Si heterojunction PDs with and without a graphene top contact in the dark and under 808 nm illumination. The light intensity is 0.11 mW/cm^2^. (**b**) *I*-*V* characteristics of Gr/PtSe_2_/ultra-thin SiO_2_/Si PD measured in the dark and 808 nm illumination with different light intensities. (**c**) The noise density spectra of our devices as a function of frequency. (**d**) Currents of PtSe_2_/ultra-thin SiO_2_/Si and Gr/PtSe_2_/ultra-thin SiO_2_/Si PDs in the dark and under 808 nm laser illumination at a voltage of 0 V. (**e**) Corresponding time-dependent photovoltaic response of Gr/PtSe_2_/ultra-thin SiO_2_/Si PD with different 808 nm light intensities at *V*_bias_ = 0 V. (**f**) Corresponding photovoltaic response of Gr/PtSe_2_/ultra-thin SiO_2_/Si PD under different incident light wavelengths at *V*_bias_ = 0 V. All the light intensities are about 0.11 mW/cm^2^.

Figure 3a exhibits the *I*-*V* curves of the PtSe_2_/ultra-thin SiO_2_/Si heterostructures with and without a graphene top contact. The SiO_2_ passivation layer can suppress the recombination current at the interface of the heterojunction and has a minuscule influence on the conduction current due to the carrier tunneling effect [37,38]. Therefore, the dark state current of the PtSe_2_/ultra-thin SiO_2_/Si device is only 16.1 pA at *V*_bias_ = −2 V, while its forward current at *V*_bias_ = 2 V is as high as 12.9 μA. The graphene top contact with high conductivity can facilitate the transport of carriers once collected at the graphene/PtSe_2_ interface, thus reducing the recombination behavior at the lateral PtSe_2_ grain boundary and improving the collection efficiency of carriers. Therefore, the reverse current of the Gr/PtSe_2_/ultra-thin SiO_2_/Si device is further reduced to 4.62 pA, and its forward current is increased to 437.3 μA, leading to a large rectification ratio of 9.46 × 10^7^ at *V*_bias_ = ±2 V. Under the 808 nm illumination (0.11 mW/cm^2^), the PtSe_2_/ultra-thin SiO_2_/Si device achieves a short-circuit current (*I*_SC_) of 0.21 nA and an open-circuit voltage (*V*_OC_) of 0.20 V. In contrast, these values of the PD with graphene contact are improved to 2.69 nA and 0.24 V, respectively. The remarkable photovoltaic characteristics demonstrate that our devices can be used as self-driven PDs to meet the demand for low power consumption [39,40]. In order to further analyze the photovoltaic effect, *I*-*V* curves of Gr/PtSe_2_/ultra-thin SiO_2_/Si device under different light intensities are shown in Figure 3b. With the enhancement of light intensity, the photocurrent of the PD is also increased due to more photo-generated carriers being generated in the heterostructure and then separated by a strong built-in electric field. In Figure 3e, the device shows a time-dependent photocurrent with good periodicity and repeatability by intermittently turning the laser on and off. Furthermore, the device maintains a large *I*_light_/*I*_dark_ value for weak light signals with different wavelengths from 375 nm to 1550 nm (Figure 3f), which demonstrates that our PD can realize broadband photoresponse with zero bias.

**Figure 4:**
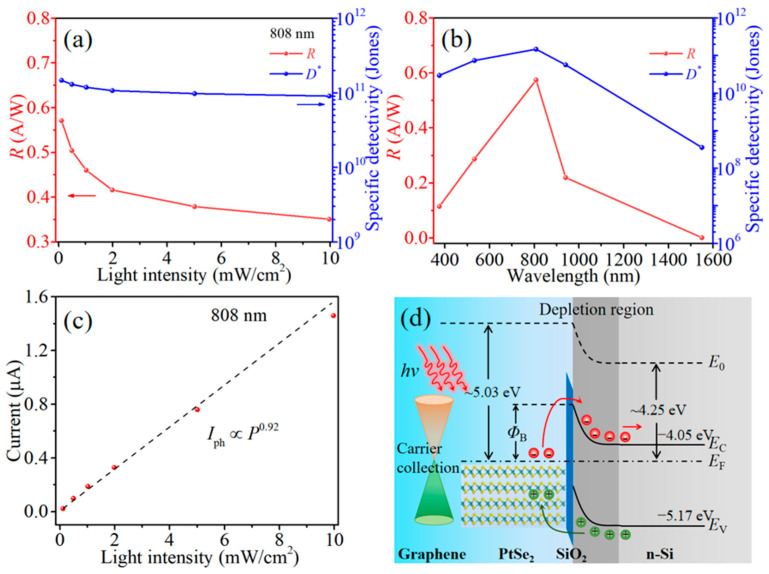
*R* and D* values of Gr/PtSe_2_/ultra-thin SiO_2_/Si PD as a function of (**a**) light intensity under 808 nm illumination and (**b**) incident laser wavelength at *V*_bias_ = 0 V. (**c**) Dependence of photocurrent on the light intensity at 808 nm illumination. (**d**) Energy band diagram of Gr/PtSe_2_/ultra-thin SiO_2_/Si device under laser irradiation.

As two important figures of merit for evaluating the performance of PDs, responsivity (*R*) represents the photoelectric conversion efficiency of the device, while specific detectivity (D*) reflects the detection ability of PD for weak light signals [41]. The *R* and D* values of the PtSe_2_/ultra-thin SiO_2_/Si heterojunction with and without graphene contact can be calculated as follows [42,43]:

(1) $$R=\frac{I_{\mathrm{ph}}}{P_{\mathrm{in}}}=\frac{I_{\mathrm{light}}-I_{\mathrm{dark}}}{P\cdot S}$$

(2) $$D^{*}=\frac{{\left(S\Delta f\right)}^{1/2}}{NEP}=\frac{{\left(S\Delta f\right)}^{1/2}}{i_{N}}R$$

where *I*_light_, *I*_dark_, *P*, *S*, and Δ*f* are the photocurrent, dark current, incident light intensity, active illuminated area, and bandwidth, respectively. The *S* value is the same as the device window area of 200 × 200 μm^2^ (Figure S5). The equivalent noise power (*NEP*) is equal to the ratio of noise current (*i_N_*) to responsivity (*R*), that is, *NEP* = *i_N_*/*R* [44]. As shown in Figure 3c, the noise current *i_N_* of the Gr/PtSe_2_/ultra-thin SiO_2_/Si PD is calculated to be 7.75 × 10^−14^ A/Hz^1/2^ at Δ*f* = 1 Hz, obviously lower than that of the PtSe_2_/ultra-thin SiO_2_/Si device (2.12 × 10^−13^ A/Hz^1/2^), which demonstrates that the graphene contact layer effectively reduces the recombination current originating from the grain boundary of the polycrystalline PtSe_2_ film. Under the same illumination condition, the carriers in the PtSe_2_/ultra-thin SiO_2_/Si device need to cross the grain boundary to be collected by the Au electrodes, leading to inevitable carrier loss. The PD with a graphene top contact provides a vertical collection path for photo-generated carriers in the PtSe_2_ film. The carriers at the graphene/PtSe_2_ interface can transport in the graphene plane toward the Au electrode, thus suppressing the recombination behavior at the lateral PtSe_2_ grain boundary and forming a larger response current. According to the photocurrent extracted in Figure 3d, the responsivities of PtSe_2_/ultra-thin SiO_2_/Si PDs with and without graphene contact under 808 nm illumination are 0.572 A/W and 0.487 A/W, respectively. Further, the external quantum efficiency (*EQE*) of Gr/PtSe_2_/ultra-thin SiO_2_/Si device can be calculated as *EQE = Rћc/qλ* = 87.5%, where *ћ* is the Planck constant, *c* is the speed of light, *q* is the unit charge, and *λ* is the wavelength of the laser [45]. Figure S6 compares the current test results of another group of devices. The PD with the Gr contact layer still shows a larger photocurrent and lower dark current, which indicates that the performance of the devices has good reproducibility. Similarly, the Gr/PtSe_2_/ultra-thin SiO_2_/Si PD obtains a D* value as high as 1.50 × 10^11^ Jones at zero bias, which is almost 3.3 times larger than that of the device without a graphene layer (4.59 × 10^10^ Jones). When the recombination behavior of photo-generated carriers becomes more significant, the recombination lifetime of electron–hole pairs will be shortened [46], resulting in a decrease in the photoconductive gain (*G*) of the device [47]. The *G* value is positively correlated with photoresponse performance; as a result, the *R* and D* values of the device will gradually decrease with the increase in light intensity (Figure 4a). To further verify the photodetection ability of the device in a wide spectral range, Figure 4b displays the relationship between the crucial performance index and the incident wavelength. For 375 nm, 532 nm, 808 nm, 940 nm, and 1550 nm lasers, the Gr/PtSe_2_/ultra-thin SiO_2_/Si PD achieves satisfying *R* values of 0.115 A/W, 0.288 A/W, 0.572 A/W, 0.232 A/W, and 1.39 mA/W, respectively. Therefore, the device obtains a large rejection ratio (*R*_808nm_/*R*_1550nm_) of ~411 at zero bias voltage. The corresponding specific detectivities of the self-powered device can be calculated as 2.97 × 10^10^ Jones, 7.42 × 10^10^ Jones, 1.50 × 10^11^ Jones, 5.71 × 10^10^ Jones, and 3.61 × 10^8^ Jones, respectively. Furthermore, the relationship between photocurrent and light intensity can be explained by the function *I*_ph_ ∝ *P^ϴ^* [48]. The fitted *ϴ* value of 0.92 is slightly lower than an ideal value of 1 (Figure 4c), suggesting that the Gr/PtSe_2_/ultra-thin SiO_2_/Si heterojunction has an efficient photo-generated carrier collection pathway and a weak charge recombination phenomenon even in the low light intensity range [49].

In order to further illustrate the working mechanism, the schematic of the energy band structure of the Gr/PtSe_2_/ultra-thin SiO_2_/Si PD is shown in Figure 4d. When n-Si is in contact with the semi-metallic PtSe_2_, due to the difference in Fermi energy level between the multi-layer PtSe_2_ film (~5.03 eV) and the n-Si substrate (~4.25 eV), free electrons will flow from n-Si to PtSe_2_ films until their Fermi levels reach equilibrium. At this time, the side near PtSe_2_ is negatively charged, while the side near the n-Si semiconductor is positively charged, generating a Schottky barrier at the interface of the PtSe_2_/Si heterojunction [50]. Therefore, a strong built-in electric field from the Si to PtSe_2_ is formed and the energy band on the Si side will bend upward, which means that our heterojunction device has self-powered photoresponse characteristics. When the wavelength of the laser is less than 1100 nm, both the PtSe_2_ and Si materials can effectively absorb photon energy to excite photogenerated carriers. Under the action of the built-in electric field, the photogenerated electron–hole pairs in the heterojunction are quickly separated even without a bias voltage. Then, the photogenerated electrons inject from the PtSe_2_ film to Si by tunneling through the ultra-thin SiO_2_ layer and are captured by the bottom InGa electrode, whereas the holes in Si can drift to the PtSe_2_ layer and be collected by the graphene layer in a vertical direction and then travel in the graphene plane toward the Au electrode, thus resulting a large photoresponse current. Notably, the existence of an ultra-thin SiO_2_ passivation layer can reduce the dangling bonds and defect states of the Si surface but has negligible influence on the migration of photogenerated carriers. When the wavelength of the laser is 1550 nm, the photon energy is lower than the inherent band gap of Si (~1.12 eV). Therefore, the carriers in the heterojunction are mainly generated from the PtSe_2_ material with zero band gap. The electrons will tunnel through the SiO_2_ passivation layer to the conduction band of Si, extending the photodetection range of Si-based PD to beyond 1100 nm.

**Figure 5:**
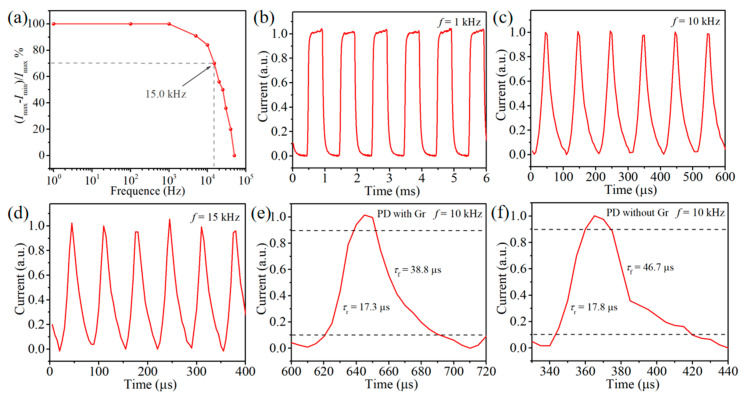
(**a**) Relative balance of (*I*_max_ − *I*_min_)/*I*_max_ versus different frequencies of the Gr/PtSe_2_/ultra-thin SiO_2_/Si device. Normalized photocurrents of Gr/PtSe_2_/ultra-thin SiO_2_/Si PD with different light frequencies of (**b**) 1, (**c**) 10, and (**d**) 15 kHz. Response speed of PtSe_2_/ultra-thin SiO_2_/Si PD (**e**) with and (**f**) without graphene material extracted at 10 kHz for 808 nm illumination.

Subsequently, the modulated optical signal is utilized to investigate the frequency response of our devices. Figure 5a depicts the relative balance of (*I*_max_ − *I*_min_)/*I*_max_ versus different frequencies of the Gr/PtSe_2_/ultra-thin SiO_2_/Si PD. When the modulation frequency is about 15 kHz, the (*I*_max_ − *I*_min_)/*I*_max_ value of the device drops to 70.7%. This large 3 dB cutoff frequency indicates that the PD has the potential to respond to high-frequency light signals. The normalized photocurrents of the Gr/PtSe_2_/ultra-thin SiO_2_/Si PD with frequencies of 1 kHz, 10 kHz, and 15 kHz are exhibited in Figure 5b–d, respectively. It is found that the device maintains a repeatable and stable fast response to the various pulsed lasers. As another important indicator of a photodetector, the response speed is determined by the rising time (*τ*_r_) and falling time (*τ*_f_). Usually, the rising time is defined as the time required for the current to rise from 10% to 90% of the saturated photocurrent, while the falling time refers to the time required to fall from 90% to 10% of the maximum photocurrent [51]. As shown in Figure 5e, by extracting from the photocurrent at 10 kHz, the rising time and falling time of Gr/PtSe_2_/ultra-thin SiO_2_/Si PD are 17.3 μs and 38.8 μs, respectively, which are faster than those of the PD without graphene contact (17.8 μs and 46.7 μs, respectively, from Figure 5f). The enhancement of response speed can be ascribed to the fact that the graphene contact with low sheet resistance provides a vertical path for the collection of carriers. Due to the carriers being extracted by the graphene layer at the graphene/PtSe_2_ interface and then transported in the graphene plane toward the Au electrode, the influence of lateral grain boundaries in 2D PtSe_2_ films on the carrier collection becomes weak. To further evaluate the practical application prospect of our devices, Figure S7a indicates that the Gr/PtSe_2_/ultra-thin SiO_2_/Si PD possesses excellent stability and synchronization for 1000 operation cycles of turning the laser on/off. The photoresponse characteristics of the device before and after one month’s storage in ambient conditions to a pulsed signal with the same light intensity are almost identical (Figure S7b). These results demonstrate that our PD has good long-term stability and durability and can be operated reliably in atmospheric environments.

**Figure 6:**
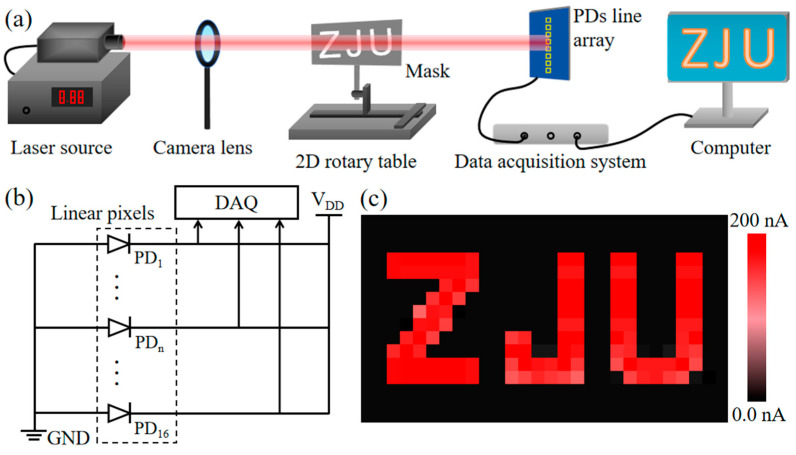
(**a**) Schematic of the experimental platform for near-infrared imaging with a Gr/PtSe_2_/ultra-thin SiO_2_/Si line array. (**b**) Equivalent circuit diagram of linear PD array used in imaging applications. (**c**) The photocurrent mapping image of the PDs array for the “ZJU” mask under 808 nm illumination.

Near-infrared light possesses substantial application potential in the fields of medical treatment, agriculture, and industrial detection [52]. In order to verify the acquisition ability of Gr/PtSe_2_/ultra-thin SiO_2_/Si PDs for high-resolution image information, we have built a measurement platform for near-infrared imaging applications (Figure 6a). The optical signal with adjustable power density is generated by an 808 nm laser source. The metal mask with a hollow letter of “ZJU” is placed between the camera lens and the PD line array. A 2D rotary table is employed to control the movement of the metal mask in the horizontal and vertical directions. As shown in the photograph of real 1 × 16 pixel units in Figure S8, the linear devices array is integrated on a customized printed circuit board (PCB) to facilitate the connection with the signal collection equipment. When the laser passes through the exposed/blocked area of the mask, the control computer obtains the corresponding photocurrent/dark-current signals of the linear PD unit recorded by a data acquisition (DAQ) system in real time. The corresponding equivalent circuit diagram is shown in Figure 6b. Under 808 nm illumination, the current mapping image of the PD line array suggests that the 16 devices have good uniformity and stability (Figure 6c). Compared with the traditional infrared sensing equipment based on InSb, HgCdTe, and quantum dots/wells, our heterojunction device array shows satisfactory near-infrared imaging capability at room temperature, which proves that the Gr/PtSe_2_/ultra-thin SiO_2_/Si PD can meet the application requirements of low cost, miniaturization, and high integration [53,54,55]. We further compare the photodetection performance of our devices with other heterojunction PDs based on 2D materials (Table 1). Obviously, the responsivity of the Gr/PtSe_2_/ultra-thin SiO_2_/Si PD under near-infrared illumination is larger than that of most other heterojunction devices even at zero bias, and it shows a fast response speed comparable to these PDs. The excellent photodetection performance of the Gr/PtSe_2_/ultra-thin SiO_2_/Si device can be ascribed to the following factors: (i) The ultra-thin SiO_2_ passivation layer reduces the number of defects and dangling bonds on the surface of Si, thus effectively reducing the dark-state recombination current of the heterojunction. (ii) The vertically stacked heterostructure provides a strong built-in electric field in the normal direction, allowing photo-generated carriers to be rapidly separated and collected by the two-terminal electrodes, consequently obtaining a fast response time. (iii) The PtSe_2_ material with a narrow band gap enhances the light absorption properties of the Si substrate and expands its spectral response range. In addition, the PtSe_2_ layer with a unique type II Dirac cone has been proven to be beneficial to the separation and transport of carriers in the vertical direction [21,22]. (iv) The introduction of a graphene top contact with low sheet resistance improves the collection efficiency of photogenerated carriers. Due to the carriers being extracted by the graphene layer at the graphene/PtSe_2_ interface and then transported in the graphene plane toward the Au electrode, the carrier recombination behavior at the lateral grain boundaries of polycrystalline PtSe_2_ films is inhibited. Therefore, the photoresponse current of the Gr/PtSe_2_/ultra-thin SiO_2_/Si heterojunction is obviously enhanced.

## 4. Conclusions

In summary, we fabricated a high-performance Gr/PtSe_2_/ultra-thin SiO_2_/Si heterojunction photodetector by cooperating with various optimization mechanisms. The ultra-thin SiO_2_ passivation layer effectively reduces the defects and dangling bonds at the Si surface. Further, the photogenerated carriers can be rapidly extracted by the graphene top contact in a vertical direction and then transported in the graphene plane toward the Au electrode, thus inhibiting the recombination behavior of charges at the lateral grain boundaries of PtSe_2_ films. The self-powered device has excellent and stable photoresponse capability in a wide spectral range. Under 808 nm illumination, the Gr/PtSe_2_/ultra-thin SiO_2_/Si PD obtains an optimal responsivity of 0.572 A/W, a high specific detectivity of 1.50 × 10^11^ Jones, and a fast response time of 17.3/38.8 μs. In addition, the PDs line array exhibits good uniformity and near-infrared imaging ability at room temperature. Our research will provide new impetus for the rise of high-performance heterostructure photodetectors based on 2D materials.

## Figures and Tables

**Table 1:** Properties comparison of our PtSe_2_/ultra-thin SiO_2_/Si PDs with and without a graphene electrode to other relevant van der Waals PDs.

Devices	*λ* @ *V*_bias_	*R* (A/W)	*τ*_r_/*τ*_f_	Spectral Range	Ref.
Si-QD/Gr/Si	877 nm @ −1 V	0.495	/	300–1000 nm	[56]
Gr/Si	875 nm @ −2 V	0.435	~1.7 ms	400–900 nm	[57]
PtSe_2_/Si	808 nm @ 0 V	0.52	55.3/171 μs	200–1550 nm	[23]
Gr/PtSe_2_/pyramid Si	980 nm @ 0 V	0.528	8.5/10.2 μs	980–10,600 nm	[28]
Gr/PtTe_2_/Si	808 nm @ 0 V	0.428	2.4/32.0 μs	808–10,600 nm	[58]
Gr/PdSe_2_/Ge	980 nm @ 0 V	0.691	6.4/92.5 μs	265–3040 nm	[59]
PtSe_2_/Gr/Si	808 nm @ −1 V	0.81	43.6/51.2 μs	375–940 nm	[60]
WS_2_/Si	980 nm @ 0 V	0.224	16/29 μs	200–3043 nm	[61]
SnSe/Si	850 nm @ 0 V	0.567	1.6/47.7 μs	300–1100 nm	[62]
WS_2_/GaAs	808 nm @ 0 V	0.527	21.8/49.6 μs	200–1550 nm	[63]
PtSe_2_/ultra-thin SiO_2_/Si	808 nm @ 0 V	0.487	17.8/46.7 μs	/	This work
Gr/PtSe_2_/ultra-thin SiO_2_/Si	808 nm @ 0 V	0.572	17.3/38.8 μs	375–1550 nm	This work

## Data Availability

Data are contained within the article.

## Supplementary Materials

The following supporting information can be downloaded at: https://www.mdpi.com/article/10.3390/nano15070519/s1, Figure S1: *n*, *k* values of the SiO_2_ passivation layer; Figure S2: Raman spectrum of Gr/PtSe_2_ heterostructure; Figure S3: TEM image and EDS mapping of PtSe_2_ film; Figure S4: AFM image of PtSe_2_; Figure S5: OM images of the PDs; Figure S6: The current of another batch of devices.; Figure S7: Stability and reliability testing of our device; Figure S8: Photograph of the imaging test platform.
