# Supplementary material for: Graphene/PtSe2/Ultra-Thin SiO2/Si Broadband Photodetector with Large Responsivity and Fast Response Time
Source: Nanomaterials (Basel). 2025 Mar 29;15(7):519. doi: 10.3390/nano15070519 (PMC11990209; doi:10.3390/nano15070519)
Supplement: Supplementary file 1 [file nanomaterials-15-00519-s001.zip › nanomaterials-3540214-supplementary.pdf]

Graphene/$PtSe_2$/Ultra-Thin SiO_2/Si Broadband Photodetector with Large Responsivity and Fast Response Time

Qing-Hai Zhu[1], Jian Chai[1], Shi-Yu Wei[1], Jia-Bao Sun[2], Yi-Jun Sun[2], Daisuke Kiriya[3] and Ming-Sheng Xu[1,*]

[1] College of Integrated Circuits, State Key Laboratory of Silicon and Advanced Semiconductor Materials, and Zhejiang Key Laboratory of Advanced Micro-Nano Transducers Technology, Zhejiang University, Hangzhou 310027, China

[2] College of Information Science & Electronic Engineering, Zhejiang University, 38 Zheda Road, Hangzhou 310027, China

[3] Department of Basic Science, and Department of Integrated Sciences, The University of Tokyo, Tokyo 113-8654, Japan

[*] Correspondence: msxu@zju.edu.cn (M.-S.X.)

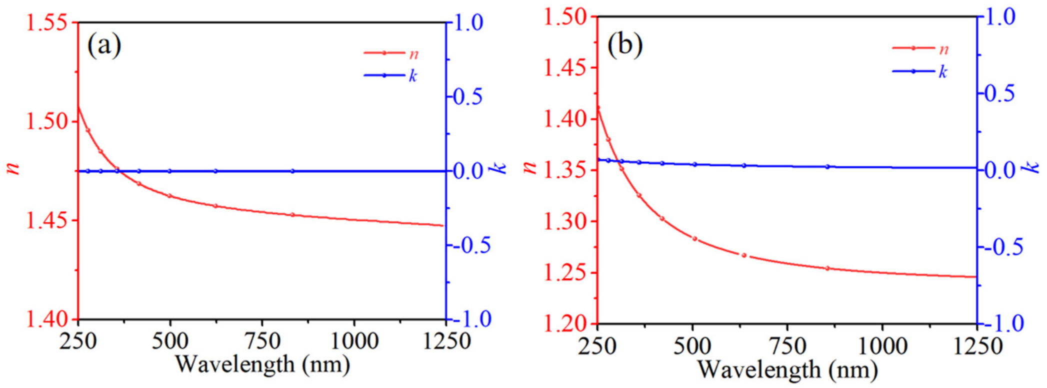

Figure S1. (**a**) Classical and (**b**) experimental *n* and *k* values of the ultra-thin SiO_2 passivation layer formed on Si substrate.

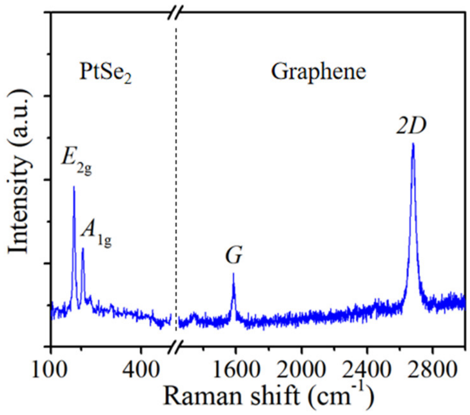

Figure S2. Raman spectrum of Gr/$PtSe_2$ heterostructure prepared by transferring graphene to the surface of $PtSe_2$ film.

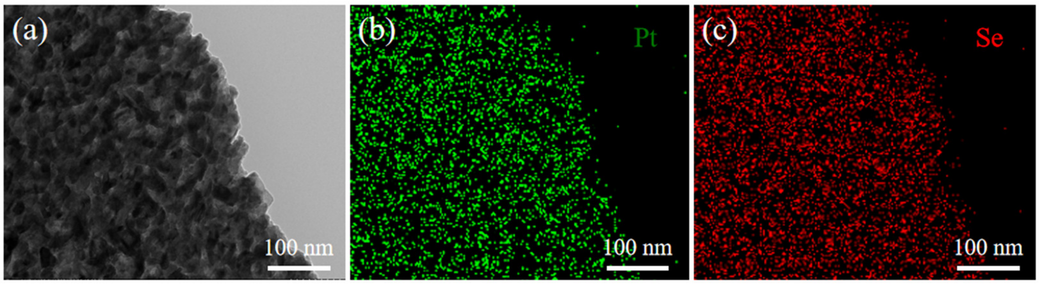

Figure S3. (**a**) TEM images of $PtSe_2$ film. And the corresponding EDS mapping of (**b**) Pt and (**c**) Se elements, respectively.

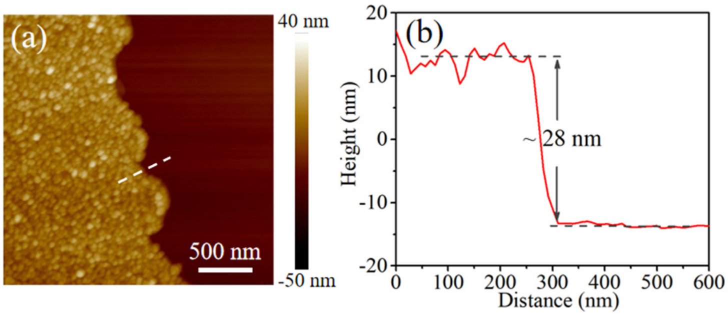

Figure S4. (**a**) AFM image of $PtSe_2$ film. (**b**) The height profile along the white line marked in (a).

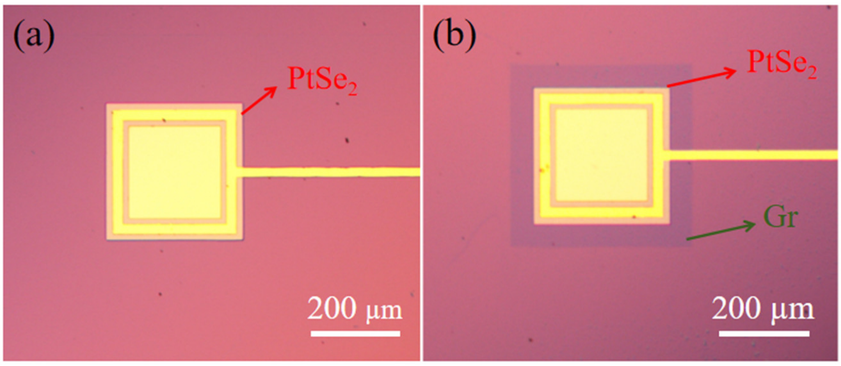

Figure S5. Optical microscope images of (**a**) $PtSe_2$/ultra-thin SiO_2/Si and (**b**) Gr/$PtSe_2$/ultra-thin SiO_2/Si PDs.

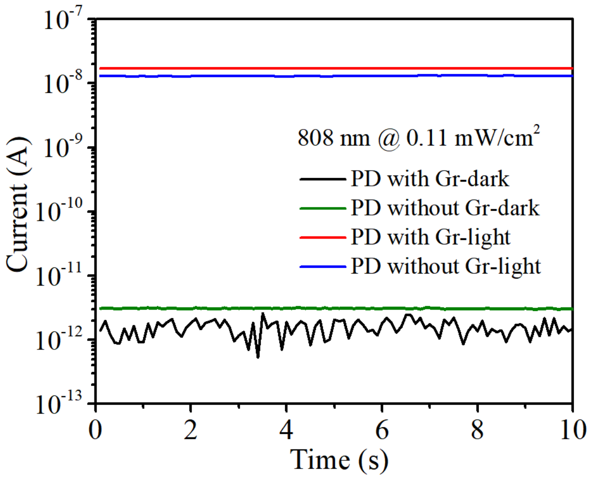

Figure S6. Currents of another group of $PtSe_2$/ultra-thin SiO_2/Si and Gr/$PtSe_2$/ultra-thin SiO_2/Si PDs in the dark and under 808 nm illumination at a voltage of 0 V.

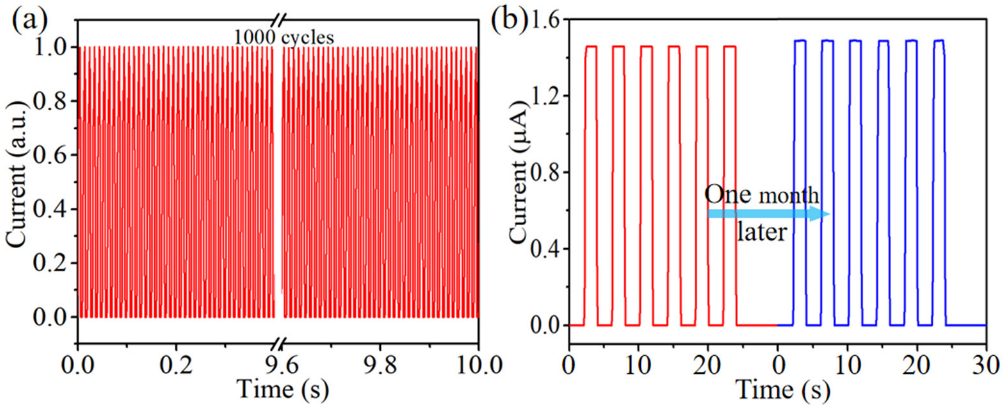

Figure S7. Time-dependent photoresponse of the Gr/$PtSe_2$/ultra-thin SiO_2/Si PD under 808 nm illumination (**a**) over thousands of cycles of operation, and (**b**) after one months' storage under ambient conditions.

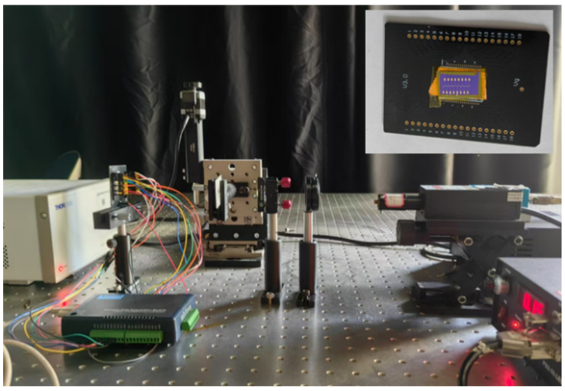

Figure S8. The photograph of the measurement system for near infrared imaging. The illustration shows the real image of a linear array with 16 pixels.
